# Responsible Innovation and Climate Engineering. A Step Back to Technology Assessment

**DOI:** 10.1007/s40926-020-00127-z

**Published:** 2020-02-19

**Authors:** Harald Stelzer

**Affiliations:** University of Graz, Heinrichstraße 26/II, A-8010 Graz, Austria

**Keywords:** Climate engineering, Geoengineering, Responsible research and innovation, Ethical impact analysis, Technology assessement, Participation

## Abstract

Much in Responsible Research and Innovation (RRI) is part of a participatory turn within the Technology Assessment (TA) and Science and Technology Studies (STS) community. This has an influence also on the evaluation of Climate Engineering (CE) options, as it will be shown by reference to the SPICE project. The SPICE example and the call for democratisation of science and innovation raise some interesting concerns for the normative evaluation of CE options that will be addressed in the paper. It is by far not clear, or so it will be argued, how much of the innovation process of CE technologies should be put in the hands of social actors and the wider public. This is due not only to special features about CE technologies but also to some more principle concerns against some features of participatory RRI approaches. Still, this does by no way mean that ethical and societal issues in the context of CE technologies should be ignored. Rather, the paper will argue that one can take a step back to expert TA linked to the evolution of approaches of ethical impact analysis in this area. This does not only lead to reconsider the emphasis on participation and democratisation of research and innovation, but also opens up for an alternative evaluative framework for CE technologies developed in the last part of the paper.

“Somewhere along the line I slipped off track.

I’m caught movin’ one step up and two steps back”.

Two lines from the song “One Step Up” by Bruce Springsteen

## Responsible Research and Innovation and the SPICE Project

In late September 2011, it was planned to send a balloon with a pipe attached to it 1 km up into the sky, to “explore the engineering challenges of using this mechanism and the accuracy/efficacy of using computer models to predict the pipe and balloon movement”(Parkhill and Pidgeon 2011, 4). This experiment was part of the Stratospheric Particle Injection for Climate Engineering (SPICE) project.^1^ In general Climate Engineering (CE) technologies are proposals for (planetary-scale) climate interventions aimed at intentionally counteracting the undesired side effects of other human activities (emitting greenhouse gas (GHG) emissions). They are diverse and vary greatly in terms of their technological characteristics and consequences. In accordance with the report of the Royal Society on CE one can discriminate between solar radiation management (SRM), aiming “to reduce the net incoming short-wave (ultra-violet and visible) solar radiation received, by deflecting sunlight, or by increasing the reflectivity (albedo) of the atmosphere, clouds or the Earth’s surface”(Shepherd et al. 2009, 79) and carbon dioxide removal (CDR), directed to “reduce the levels of carbon dioxide (CO2) in the atmosphere, allowing outgoing long-wave (thermal infrared) heat radiation to escape more easily”(Ibid 76).

As the injection of particles in the stratosphere is the most prominent SRM option the balloon-experiment of SPICE focused on the engineering part of a potential delivering mechanism. However, it is important to know, that for SRM even very small-scale field experiments are still on hold due to more general ethical concerns. From the start of the current debate on SRM in 2006 there has been a widespread acknowledgement in the scientific as well as the political community that research and development as well as the possible deployment of such options raise many important ethical, societal, and political issues. These concerns also caught up with the SPICE project. As a result the balloon never got off the ground. The trial was first postponed by one of the main funders of the study, the UK Engineering and Physical Sciences Research Council (EPSRC). Funding for SPICE was based on a ‘stage-gate’ review process for each discrete stage of research and development, including a reflection on risks, uncertainties and impacts surrounding the test, but also on SRM more generally. The stage-gate panel, which included atmospheric scientists, engineers and social scientists, as well as an adviser to an environmental NGO, evaluated the SPICE project by five criteria for responsible innovation. They were:

“the test-bed deployment was safe and principal risks had been identified, managed and deemed acceptable; the test-bed deployment was compliant with relevant regulations; the nature and purpose of SPICE would be clearly communicated to all relevant parties to inform and promote balanced discussion; future applications and impacts had been described, and mechanisms put in place to review these in the light of new information; and mechanisms had been identified to understand public and stakeholder views regarding the predicted applications and impacts.” (Macnaghten and Owen 2011).

Even though the panel found that the first two criteria had been met by the efforts of the SPICE team, it concluded that more was required on the remaining three (Stilgoe et al. 2013). A revised communications plan to inform further public debate was demanded as well as more communication with stakeholders, including a review of the risks and uncertainties of SRM also regarding the social, ethical, legal and political dimensions. However, the increased participatory activities led to an open petition signed by more than 50 non-governmental and civil-society organizations, demanding the cancellation of the project. After one scientist, who was involved in the project, had submitted as a co-author a patent for a technology similar to that used in the project, the decision was made to finally call off the field test in May 2012 (Cressey 2012). John Shepherd, chair of the Royal Society’s geoengineering group said in an interview with The Guardian:

“This shows how commercial and financial interests can complicate the management of research on geoengineering, especially SRM technology, even if everyone agrees that it is safe. The project team have done the right thing, but this is an issue that needs to be explored in depth with stakeholders.”^2^

One could say that the genie that got out of the bottle with SPICE is still around. The cancelled test had not only developed a symbolic meaning and opened up a broader reflection and deliberation on SRM, but also resulted in a slowing effect on field tests in this area of research.^3^ It took until 2020 for the Stratospheric Controlled Perturbation Experiment (SCoPEx) to move SRM research out of the lab and into the stratosphere. The plan is (in early 2020) to send up a steerable balloon 20 km above the southwest of the United States. It will release small plumes of calcium carbonate (100 g).^4^ Turning around and flying back the instruments on the balloon would then show how the particles disperse (Tollefson 2018). The idea of SCoPEx has also triggered lively discussions within the CE community. As with SPICE the underlying dispute is not about the possible consequences of the experiment itself – which are environmental benign –, but rather if SRM research is ready for field tests, or if these should be postponed until adequate governance (on an international level) is in place and further discussions on CE in general are conducted with stakeholders and with the wider public.

The SPICE ‘misery’ as well as the further debates on CE field tests are very good examples of the work of the new emerging field of responsible research and innovation (RRI). This is also evident by the so-called Oxford Principles for CE governance (Rayner et al. 2013). These principles not only put forward the demand to regulate CE as a public good, but also call for public participation in CE decision-making, wherever possible already for research activities by those affected. Other aspects of RRI are as well reflected in these principles. Principle 3 asks for the disclosure of CE research and open publication of results, and Principle 4 calls for an independent assessment of impacts of CE research. Such an assessment should not only address environmental but also socio-economic impacts, including mitigating the risks of lock-in to particular technologies or vested interests. Principle 5 calls for governance before deployment. These principles were adopted by the UK House of Commons Science and Technology Committee and subsequently approved by the Scientific Organising Committee at the Asilomar International Conference on Climate Intervention Technologies. The implementation of these principles was supposed to be part of stage-gate processes, similar to the one in the SPICE project and intended to stimulate discussions between scientists, policy-makers, civil society groups and citizens on overarching societal values for the guidance of CE governance.

Furthermore, Stilgoe et al. (2013) developed their RRI framework in connection with CE research, especially within the SPICE project. Based on this project they have articulated and explored four integrated dimensions of RRI: anticipation, reflexivity, inclusion and responsiveness. In their account they aim for the inclusion of a wider moral responsibility to society into the research and innovation process and call for a ‘democratisation’ of science and innovation, extending to a wide range of (social) actors and the wider public. This should help to create a new type of knowledge for the social, political and ethical implications. The aim of their RRI approach is to take care of the future by the development of a collective stewardship.

The link between CE research and RRI as well as the demand for public engagement is indeed important and valuable due to the potential large-scale deployment and consequences of such technologies. Still, the SPICE example as well as the call for democratisation of science and innovation by Stilgoe et al. raise some interesting questions. As it will be argued it is not clear of how much of the innovation process of CE technologies should be put in the hands of social actors and the wider public. At the one hand this is linked to special features about CE technologies, like their early research state, the associated uncertainties as well as the ideological background assumptions considering their potential deployment. On the other hand one can also put forward some more principle concerns against some features of participatory RRI approaches. The failure of the SPICE project shows that the participatory turn in RRI may cause some set-backs for research as well as for our evaluation of different options. Holding on to the need for ethical analysis the paper puts forward the idea to take a step back to expert TA. Rather than ‘opening up’ the debate such an approach is in favour of ‘closing it down’, in order to cut through the messy, intractable and conflict-prone diversity of views and to develop prescriptive recommendation to assist in decision-making processes. If it is correct, that RRI originates from TA, this approach does not renounce the close relation to a wider understanding of RRI. However, as it will be argued, one could reconsider the emphasis on participation and democratisation of research and innovation, at least at the current state of development of CE technologies and options. To make this argument it is important to first develop a better understanding of RRI. Where does it come from, and what are the main aims and approaches? What specific challenges do these approaches face? This will lead to the consideration of alternative forms of ethical analysis that will be introduced to show the evolving field of ethical impact analysis. Based on these alternatives the paper will end by developing a specific evaluative framework for CE technologies, able to deal with ethical and societal questions in a more nuanced way. Let us therefore start by the origins of RRI and then look at some basic challenges, before we return to climate engineering and search for an adequate evaluative framework.

## Origin and Challenges of Responsible Research and Innovation

The origin of RRI can be found in interdisciplinary Technology Assessment (TA). TA was and is used for the evaluation of emerging technologies in order to provide knowledge or foresight and thereby allowing better decision-making (Grunwald 2015). TA was initially conceptualised as policy advice to support policymakers by exploring political measures, adequate regulation, reflecting priority-setting in research funding, and by developing strategies towards sustainable development. The main task was seen in its early-warning function in order to enable political actions (Grunwald 2011a). As an interdisciplinary approach, TA observes trends in the technology sector and analyses the associated social developments and gives recommendations for political and economic action, which serve to avoid risks and identify opportunities. It has found its way into many areas of society and various thematic complexes, e.g. environmental, energy, health or safety issues.

Soon TA has split up in many different approaches like ‘Constructive Technology Assessment’ (CTA) (Schot and Rip 1996), ‘Real-Time Technology Assessment’ (Guston and Sarewitz 2002), or ‘Participatory Technology Assessment’ (PTA) (Sclove 2010). The turn to CTA is also due to the uptake of the social constructivist paradigm developed during the 1980s, which emphasises on the ‘shaping of technology’ according to social needs and values. Within CTA, two elements already moved to the fore: a broader basis for technology development by the inclusion of more aspects and more actors, and, second, a better understanding of the dynamics of technology development and its embedment in society, also in order to steer innovation. This was supported by the development of Science and Technology Studies (STS), moving towards a more reflexive co-evolution of science, technology, and society (Fisher and Rip 2013, 176). The idea to influence, guide and direct the development and innovation processes has created the new emerging field of RRI or in its more applied form: ‘Responsible Innovation’ (RI).

RRI sets forward the hope to be able to shape technology according to social values, which would also minimize problems of rejection or conflict. RRI calls for an intense inter- and transdisciplinary approach by integrating elements from: applied ethics, which addresses the moral dimension (normative reflection on responsibilities, values, norms and principles); philosophy of science, which takes care of the epistemic dimension; TA, which provides input on impacts and its experiences on assessment procedures, actor involvement, foresight and evaluation; as well as social sciences such as STS and political science for a better understanding of regulation and policy development (Grunwald 2011a). New approaches towards RRI see the balancing act of the positive and negative effects of introducing new technology not as the responsibility of political processes but rather distribute “this responsibility *throughout* the innovation enterprise, locating it even at the level of scientific research practices”(Fisher and Rip 2013, 165). The particular distribution of responsibility has also consequences for the governance of the respective field and relates to questions concerning the purpose, motivation, goal and direction of innovation and the provided social and political framework (Stilgoe et al. 2013). RRI is responsive towards new knowledge and emerging perspectives, views and norms, by having the possibility to adjust the courses of action while also recognising the insufficiency of knowledge and – as pointed out by Collingridge (1981) – control (Stilgoe et al. 2013).

New approaches of RRI are constantly developing and challenging the traditional view on innovation. As pointed out by Timmermans and Blok (2018) this still influential traditional innovation paradigm, shown in the understanding of innovation processes by Schumpeter, is based on certain ontological assumptions: such as understanding innovation predominantly as technological innovation, a focus on economic aspects and the guidance by market-forces. These ontological assumptions correspond to a capitalist worldview, valuing innovation in economic terms and leading to the neglect of considerations for societal or ethical values beyond economic ones. From different RRI approaches this traditional innovation paradigm has been criticized for seeing innovation as something solely positive and inherently good, taking only little notice of potential negative side-effects and unintended consequences. The need to take all possible consequences into account also questions the solely focus on technological innovations and economic consequences (e.g. creating prosperity or improving the economy) of the traditional innovation paradigm. This not only leads to the ignorance of other forms of innovation, like social or service innovations, but also to the neglect of particular considerations for societal or ethical values beyond economic ones. Furthermore, the traditional paradigm is linked to a top-down regulative framework by normative and legal constraints. The embedment of technologies in society also questions the narrow traditional perspective on actors associated with innovation, since it is often reduced to those who are all more or less concerned with competitive advantages gained by innovation.

New approaches towards RRI are able to escape some of these shortcomings. They consider not only different forms of innovation, but also allow for societal and ethical issues to play a role in the assessment of the innovation processes. As pointed out above, they attribute responsibilities throughout the innovation enterprise. Here responsibilities are closely linked to the possibilities of actors to influence actions and decisions in the respective field. It therefore depends on the distribution of the capabilities to act and decide in the field considered. Furthermore, it allows for the assessment of technologies at an early development stage by anticipating and reflecting societal and ethical aspects of the innovation. RRI has been described as a framework to address societal dimensions of science and technology, understood as a ‘forward-looking responsibility’ responding to new emerging knowledge and acknowledging the uncertainty and limited control inherent to innovations (Sonck et al. 2017). It combines “(ethical) acceptability, sustainability and societal desirability of the innovation process and its marketable products” (Von Schomberg 2013, 63). RRI attributes a prominent role to ethics and is explicitly aspirational in character by aiming not only to avoid negative consequences, but rather at realizing ‘the right impacts’ (Noorman et al. 2017). Stahl et al. (2017) understand RRI as a meta-responsibility shaping, maintaining, developing, coordinating and aligning existing and novel research and innovation related processes, actors and responsibilities in order to ensuring acceptability, desirability and sustainability of research processes. The early engagement in the design process is based on responsiveness as the action element of RRI. This can help the moral as well as societal legitimization of innovations by including the perspectives of a wide variety of stakeholders. RRI is often seen as a process, involving interaction between innovators and societal stakeholders, who should be mutually responsive to each other. This requires reciprocity, learning from each other as well mutual trust among actors with very different needs and interests (Nielsen 2016).

Noorman et al. (2017) distinguish between RRI approaches in terms of substantive norms regarding the outcome and those in terms of procedural norms regarding the process. Within a position like the one advocated by Von Schomberg (2013) responsiveness is based on normative ‘anchor points’ like gender/equality and diversity, social justice and inclusion or sustainability. Here the reason for stakeholder involvement is driven by the idea to obtain better results in the innovation process (Stahl et al. 2017). Beside such normative based approaches there are also procedural approach that see stakeholder involvement as a demand in itself often linked to a democratic orientation. Here the outcomes are less fixed while the emphasis is put on the inclusiveness of the process. Rather than taking the normative anchor points as a given the deliberation process aims to arrive at mutual understandings and shared goals, values and expectations. Especially in situations of high ambiguous uncertainty existing normative guidelines may not adequately represent stakeholder perspectives and capture societal concerns. For new and emerging technologies broader and more inclusive reflection on the guiding norms could therefore become vital for the acceptability and overall success of the innovation (Sonck et al. 2017).

Even though the last years have seen the development of quite some approaches for the engagement of stakeholders, not only in the policy-oriented framework but also in cooperate settings (eg. Stahl et al. 2017; Noorman et al. 2017) there are still some constrains that should be considered before deciding to ‘open up’ the debate on certain emerging technologies.^5^ A first challenge is the possible lack of consensus among stakeholders on what the real problem is as well as on how to judge alternative solutions. Multiple stakeholders can differ widely in respect to their view on the problem situation as well as the societal and ethical aspects that should be taken into consideration during the innovation process. Because of these differences between various stakeholders concerning the definition of the problem situation, as well as the motives, values and goals of collaboration, their involvement in development and innovation processes is liable to failure (Blok and Lemmens 2015, 22–23). Furthermore, in science and innovation we must often deal with forms of shared or collective responsibility by different actors. As pointed out by Stilgoe et al. (2013), this can lead – based on the complex nature of these systems – to what Ulrich Beck called ‘organised irresponsibility’.

A second problem can be found in fundamental power imbalances between stakeholders. Blok and Lemmens (2015, 23) refer to the ‘push and pull’ effects of actors which are more powerful than others in the definition of the problem and the objectives of the innovation process. Just by involving different stakeholders one cannot expect that all of them will have an equal say. Furthermore, there are also possible instrumental imperatives by power asymmetries, which lead to “implicit, but potentially powerful, conditioning pressures”(Stirling 2006, 98). Even though sometimes this will be employed to willingly manipulate the participatory processes, in many cases those asymmetries are the result of constraints of time, resources, power and knowledge. There may also be a lack of commitment by some stakeholders to get or stay involved (Sonck et al. 2017). In other cases the identification of the relevant stakeholders may be difficult or they may be distant in time, space or discourse. Time and financial constrains can further complicate stakeholder involvement (Noorman et al. 2017).

A third problem is the finiteness of “transparency” and “reciprocity” between stakeholders within innovation processes. With reference to the Collingridge control dilemma, Blok and Lemmens (2015, 25) show that transparency and interaction with multiple stakeholders does not make innovation processes any easier to manage. If responsible innovation processes are characterized by information asymmetries, the presumed mutual responsiveness to stakeholders becomes questionable. Such asymmetries may even be used to support misleading claims about the characteristics or capacities of innovations under development in order to attract social and ethical legitimacy.

A fourth problem is based on a possible lack of legitimacy. The participation and democratization of the innovation processes need new types of institutional arrangements that may replace legitimate legal and democratic institutions and yet have to prove their legitimacy. Furthermore, the more active involvement of stakeholders and the public “may not be able to sustain the proper checks and balances and due diligence that would be required to legitimize RI”(Timmermans and Blok 2018, 27).

Challenges can also arise on the epistemological level due to factors like the inherent complexity, uncertainty and unpredictability of technological innovations. The subject of responsibility is often only known to a limited degree. Statements about impacts and consequences of research and new technologies usually show a high degree of uncertainty. In many cases, the body of available knowledge only consist of assumptions about future developments and the proper methods and procedures to assess the rationality behind uncertain images portrayed for the future especially on the conditions that must be fulfilled for this future to come into existence. While reliable knowledge about those future developments usually is hard to achieve, also expectations, fears, concerns and hopes play an important role. The inevitably high degree of these expectations constitutes a fundamental problem for responsibility debates about far-ranging future developments in science and technology (Grunwald 2011a). Blok and Lemmens (2015, 28) summarize the epistemological problems as follows:

“If the output of responsible innovation processes is characterized by a fundamental uncertainty, which means that our knowledge of the impact of our innovations is not only limited but principally insufficient, the presupposed ‘foresight’ of responsible innovation becomes questionable. In other words, our knowledge is principally insufficient to assess the impact of innovation processes and there will always be unintended consequences of our innovations which can be harmful.”

## Back to Technology Assessment

It is of course an open question if practice should be lifted to theory or theory adapted to make it more practicable and realistic in different contexts (Noorman et al. 2017). Here I will not engage further in this question but rather suggest that problems with inclusivity and responsiveness of current RRI approaches, could speak in favour of ‘closing down’, rather than ‘opening’ the debate. The process of opening a debate places special emphasis on marginalised perspectives, neglected issues, ignored uncertainties and alternative options. Even though it is important to reflect and include implications of different sources of information, divergent social values and conflicting interests, this can lead to disparate interpretations of the available evidence. In focusing on these aspects, the advice delivered to decision makers is ‘plural and conditional’ (Stirling 2006; for a detailed account of such an approach for CE see Macnaghten and Szerszynski 2013). The approach put forward in the rest of this paper focuses rather on ‘closing down’ with the aim to cut through the messy, intractable and conflict-prone diversity of views in order to develop prescriptive recommendation to assist in decision-making. “In pursuing this aim, the focus is on identifying the salient viewpoints, finding the priority issues, highlighting the most likely outcomes and so determining the ‘best’ options” (Stirling 2006, 101). Therefore, it will be argued that in the case of CE we should go back to TA and its early-warning function. This is in line with the aim of TA as outlined by Bechmann et al. (2007, 19): “to investigate the combination of ecological, social and economic conditions, concentrate them for political decision makers under the perspective of sustainable and environmentally sound development and present them ready for decision making. […] This requires an interdisciplinary, integrative approach in research which, at the same time, must realise that this knowledge must be organised and processed in order to be useful for action and for decision making.”

This stands in opposition to process oriented RRI approaches as well as closely related trends within the STS community (Timmermans and Blok 2018). As discussed above the drive for participation and democratic inclusiveness may face problems of their own such as the possible power imbalance and information asymmetry between different stakeholders as well as questions of feasibility and legitimacy. Participatory approaches must not always have positive effects for certain stages of research. Especially when dealing with a ‘wicked problem’ like climate change we are confronted with an ambiguity of the definition of the problem situation and the vast differences in the evaluation of alternative climate policies.^6^ It is not clear how stakeholder engagement will help to overcome those conflicts that are often not based solely on an information gap, but also on deeper conflicts of interest or differences in underlying values, norms and principles. Furthermore, the inclusion of new actors would raise important issues regarding its feasibility, their capacity and motivation, and the ability and motivation of researchers and innovators (Timmermans and Blok 2018).

Going back to TA, however, puts forward the question, if it is able to deal with the underlying societal and ethical questions of emerging technologies or if it falls prey to the same shortcomings as the traditional innovation paradigm. It is clear that it cannot do without ethics because values, norms and principles are constitutive factors for TA. It were exactly these normative deficits that lead to the turn towards stakeholder involvement and/or public participation. However, in the development of these inclusive accounts at some point the democratic or procedural elements can endanger to outweigh substantive ethical arguments (Grunwald 1999, 170–172). This is problematic as the social state of a de facto approval or disapproval does not entail any considerable normative force. After all, a majority may be wrong. One could argue, that a key idea of participatory approaches is that it is a joint and inclusive decision, not that it helps us to make the right decision.^7^ However, if we are at an early innovation and development stage it might be more important to undertake the research that can provide us with the necessary information do make well reflected decisions. This does of course include ethical and societal concerns. Still, they can be viewed from the perspective of experts, and do not need to depend on inclusiveness (see bellow). Besides the stronger emphasis on participatory elements and ethical aspects in different developments in TA, recent years have also seen the evolution of specific TA ethics models, that are able to provide such input.

One early form can be found in Rational Technology Assessment (RTA), with a focus also on unintended impacts (based on interdisciplinary expert discussions) and ‘normative acceptability’. Traditional TA has usually weighed scientific and procedural criteria higher than ethical values. RTA is a reaction to precisely this lack of values in TA. With the means of practical rationality, it sets out to examine different ethical concepts that could lead to conflicting views on technologies at an early state (Gethmann 1999). At a time when the momentum in TA was almost exclusively on the reinforcement of participative elements, the differentiation between factual acceptance and normative acceptability made an important contribution to the discussion in TA concepts. However, the lack of influence of this approach was mostly due to the missing participatory elements (Bechmann et al. 2007).

Ethical TA (ETA) can be seen as a further step in this development. As the name indicates, it aims at including ethical impacts into the assessment of technologies with the purpose “to provide indicators of negative ethical implications at an early stage of technological development”(Palm and Hansson 2006, 543). In general, ETA is an approach that describes how ethicists can be systematically involved in technology development throughout the entire process. ETA therefore rests on an interdisciplinary setting that allows for a dynamic process between scientists, developers and ethicists. It is not an assessment done in isolation, but rather a complement to existing TA processes with a realistic focus on the ethical implications, based on what is already known about the technology. From a methodological point of view, ETA does not strive to establish a coherent axiology, but rather uses checklists that list ethical issues and questions for reflection, supplemented by the involvement of various interest groups, in order to get a better grip on their understanding of the underlying societal problems (Nielsen et al. 2015, 4 & 11–12; Palm and Hansson 2006, 543–44 & 549–51).

Ethical Impact Assessment (EIA) has some similarities to ETA and is used for the ethical evaluation of technology development projects. EIA investigates how technologies may be used in the future as components of a larger technological framework.^8^ It therefore extends towards a broader assessment of emerging technologies. Compared to ETA, it aims at a higher level of clarifying the normative issues at hand. EIA attempts to find workable conceptualisations of ethical impacts and the ethical values and principles which apply to them, to assess the relative importance and the likelihood of the occurrence of ethical impacts and to locate potential value conflicts and, where possible, to resolve them. A first step often consists in a threshold analysis providing answers whether considerable ethical impacts can be expected from research or the implementation of an emerging technology (Reijers et al. 2016). If ethical aspects are shown to be relevant, this is followed by an impact identification stage which maps the ethical impacts that might occur in the context of the new technology and puts these in a temporal perspective (anticipating short/medium/long-term impacts). Knowledge about possible ethical impacts can be gained by consulting existing literature or ethical impact assessments of similar technologies. However, often a multitude of perspectives will be needed to assess both what kind of impacts can be deemed ethically problematic and to know what the likelihood of an occurrence of those impacts is. For this reason, the ethical impact identification stage often includes approaches for stakeholder involvement and the consultation of experts. In this way, it attempts to look beyond what is directly known about the technology.

In contrast to ETA, EIA retains some prognostic elements. It sets out to predict (ethical) consequences by using methods from the field of forecasting and foresight (e.g. scenario methods, Delphi panels, expert consultation) to construct a possible picture of the future (Nielsen et al. 2015). This allows to identify likely, plausible or possible developments which are then investigated from an ethical point of view by first identifying and evaluating ethical issues (Brey 2017). Even though foresight activities can prove to be challenging, they are an important part of trying to face the problem of uncertainty in ethical approaches towards emerging technologies (Pereira et al. 2007). Including foresight does not mean ignoring questions of uncertainty. Quite to the contrary, it can be linked to the active search for possible knowledge gaps.

Grunwald has discussed this search under the topic of “explorative philosophy”(Grunwald 2011b, Ch. 10). Such an approach is epistemologically aware when assessing the degree of rationality behind images of uncertain futures based on developed and applied methods and procedures. Accordingly, it is necessary to examine the cognitive and the evaluative content of the prospective knowledge used in debates to describe the subject of responsibility. This is based on an epistemological “deconstruction” of prospective statements. For the components of knowledge and the knowledge deficits involved, their respective premises and how they are combined into coherent visions of the future must be identified. An important aspect of this is to examine the conditions for the realisation of these futures and the associated timelines of their possible realisation. In this context, the vision evaluation approach has been proposed to discover the epistemological and ethical foundations of a visionary future (Grin and A. Grunwald (Eds.). 2000). The objective is to uncover the epistemological and normative components of future statements in order to allow for better informed and rational opinion formation, evaluation and decision-making on the assignment of responsibilities. In this way, vision evaluation can contribute to ethical and responsible reflection by helping to avoid the problem of ‘mere possibility arguments’(Grunwald 2011a). As pointed out by Hansson, such arguments are not enough to guarantee responsible decision-making as any decision or option may have catastrophic unforeseen consequences. Policies that attend seriously to all such arguments “would make us hostages to the constructors of fanciful scenarios” (Hansson 2013, 89). It is essential that the status of the available knowledge is critically reflected on from an epistemological point of view.

## Evaluating Climate Engineering

Going down this road would also change our discussion and evaluation of CE. It would not only lead us away from some of the problems and shortcomings linked to the participatory RRI approaches but also from the highly ideological debates still dominating opinions on CE. Arguments against CE are often not based on the impacts or features of specific technologies, but rather on the general assumption that committing to CE is a symptom of our unwillingness to address climate change in a sustainable and responsible way (Gardiner 2010, 2013). CE technologies are considered as leading to prolong or to lock us into the social and economic order of today, an order by many perceived as unsustainable and unjust. This would leave the underlying problem of anthropogenic climate change, the careless overconsumption of natural resources (including GHG sinks), consumerist life-styles or GHG intensive agriculture, energy or consumer goods production and population trends unaddressed (Gardiner 2011; Macnaghten and Szerszynski 2013; Corner and Pidgeon 2010). There is a considerable distrust against understanding CE as a technological innovation, as such options could leave aside possible solutions on the social level. Furthermore, CE has been linked to a deep-rooted habit of Western cultures to solve problems with technology, rather by responding more directly to the failure of people to behave in an appropriate way (Borgmann 2012). The techno-fix framing is therefore often used negatively by connoting an inadequate and morally problematic solution for the underlying behavioural problem (Scott 2012 and 2013; Corner and Pidgeon 2010).

Without doubt these arguments are important starting points for the debate that can link CE to deeper questions concerning our current social and economic order as well as our responsibility towards a sustainable future. However, they are not inclusive and can be contested, just like other arguments like the moral hazard or hubris one (Stelzer 2017b). Furthermore, the ideological background assumptions underlying these debates can block a more differentiated evaluation of developing CE options based on interdisciplinary research and on more accurate criteria to inform our judgments (Tuana et al. 2012). It seems that current arguments often focus on what Brey (2012) calls the technology level at which a particular technology is defined independently of the different options of applications that may result from it. In line with the missing differentiation of different technologies that is often witnessed in CE debate, this level refers therefore to a collection of options that are related to each other by certain features, ignoring important differences. As pointed out in Brey’s anticipatory technology ethics (ATE), for a fuller understanding of a technology we need to move from the technology to the artefact or procedure level. Here we will have to deal with the inherent characteristics of a certain option, the unavoidable consequences in most or all of its uses, and its risks as well as other features that could make it morally controversial. This will also lead us from the identification stage in which ethical issues are identified to the evaluation stage in which they are evaluated (Brey 2012).

By integrating social and ethical concerns and by guiding our way through the evaluation of uncertain futures, taking a step back to an ethical informed TA may be able to provide important insights when it comes to solutions to wicked problems like climate change. The integration of ethics at an early stage should help to anticipate and preempt moral concerns (van den Hoven 2013). Even though already RTA was criticized for only partly meeting the claim of being an integrated evaluation tool by restricting itself to an interdisciplinary expert TA (Bechmann et al. 2007), regarding CE the following evaluative framework will rest on an expert driven principle-based ethical approach, rather than a stakeholder-based ethical analysis (Reijers et al. 2016). Stakeholder-based ethical analysis – aiming for the direct inclusion of stakeholders into the processes – may not only be expensive and time consuming, it may also be biased and subject to the problems described above. Furthermore, a principle-based ethical approach is closer to the work of ethicists and other experts in the field who often engage in stakeholder-based ethical analysis only to a limited extent. Such a principle-based analysis involves the identification of interest groups to whom specific interests, rights and obligations are assigned. However, these assignments are made without their contribution. Of course, this also has its drawbacks as it is based on less reliable data, as values and interests are projected onto stakeholders. It is also less democratic and can introduce distortions and the preferences of analysts that remain uncontrolled by others.

Nevertheless, even a principle-based ethical analysis takes us way past the reductionism of the traditional techno-economic innovation paradigm towards the consideration of societal, ethical and ecological values. What counts is not what best serves the interests of individuals, groups and organisations, but rather refers to the respect of the rights of others, the avoidance of harming them, the furthering of their well-being or the distribution of benefits and burdens (Brey (2012) also uses these dimensions for his ATE approach). The balancing of interests results in a balancing of ethical principles which determine when, if at all, certain harms may be justified or rights may be violated, and how rights must be balanced against each other and against harms and benefits to well-being. Ethical TA allows to take into account explicitly ethical issues, where a technological option potentially violates moral principles, values or norms, as well as intuitive ethical issues, “where a technological option has certain characteristics or implications that intuitively feel morally problematic or controversial, even though it is not immediately clear how and whether the option violates any moral value or principle”(Reijers et al. 2016, 35).

This differentiation is of importance because our evaluation of emerging technologies is often not solely linked to their potential impacts which are known to us only to a certain degree, but to special inherent features. For example, controllability is a key aspect of responsibility and for the public acceptability of risks. It refers to certain characteristics of a technology, e.g. reversibility, defined as the ability to ‘switch off’ a technological program and have its effects terminated in a short time. Reversibility therefore applies to more than just the ability to end deployment. It also concerns questions about possible rebound effects or time lags of effects even after ending implementation. E.g., the injection of aerosols in the stratosphere comes with a termination effect because a failure to maintain the aerosol counterforcing could result in an abrupt and potentially very damaging warming (Vaughan and Lenton 2011). Reversibility is not to be reduced to environmental consequences. The abandonment of certain technologies could also face strong resistance due to vested interests based on the investments made in the construction and maintenance of the physical infrastructure or the use made of such technologies to keep up the status quo (Preston 2016). A further aspect of controllability is flexibility, as it enables us to react to unforeseen consequences. Also ‘encapsulation’, understood as the amount of ‘foreign material’ that is released into the environment or the possibility to contain the engineered system, refers to controllability (Shepherd et al. Shepherd et al. 2009). The importance of this aspect to CE is mostly based on the scale and the duration of the manipulation of the natural environment and ecosystems. This is also related to a further interesting aspect of the discussion of CE: scalability. Here an important question is whether a technique allows for sub-scale testing and gradual deployment to full-scale. The novelty of the involved processes might also be considered in this context as well as questions of detection and attribution.

Also, constraints that refer to natural aspects, as well as to social, political, and economic issues can help to evaluate normative aspects of different options. For TA, it is of course one of the main questions whether an option is at all technological feasible. Also, the economic viability and social and ecological sustainability remain to be determined (Shepherd 2012; Welch et al. 2012; Stelzer 2017a). E.g., many CDR options would only be economically sound if the uptake of CO_2_ were cheaper and/or more efficient than limiting emissions in the first place (Schellnhuber 2011). Political feasibility may depend on the expected costs and benefits of a technology but also on its environmental consequences and the risks involved. It is also linked to public acceptability and the conflict potential of a technology. E.g., conflicts could result from the unilateral deployment of some CE technologies, the potentially uneven distribution of costs and benefits, trans-boundary effects, problems of tracing consequences and assigning responsibilities, or competing interests of regional climate control. Questions of procedural justice and governance issues also fall under this category. A further characteristic worth mentioning is sustainability that connects with controllability and feasibility, but adds questions to the long-term consequences (including life-circle analysis) and the use of resources.

For a basic understanding of the characteristics of a technology we must rely on the scientific results such as impact analyses of the natural sciences, technological studies of engineering or societal aspects brought forward by SSH research. For at least some CE technologies (especially for CDR) we can reach back to our experiences with already existing and used technologies. Lately much work has been done on basic questions concerning different CE options, creating a better picture of effects and side-effects, of the involved chemical and biological processes, energy resources, and demands on land and infrastructure, just to name a few. Still, even though recent years have shown an increase in our understanding of different technologies, it seems unrealistic that any time soon can we come up with exact estimates or provide a full understanding of all possible consequences of deployment. We are therefore limited to rely on expected effects and risks of certain options or deployment scenarios.

For some of the inherent features of CE technologies mentioned above generic approaches based on conceptual analysis and empirical observations are suited in order to identify and consider necessary conditions for their realization or potential impacts. However, not all of the expected impacts and risks are independent of how those options will be used. This indicates the need for anticipatory approaches that combine ethical analysis with various foresight, forecasting or futures studies techniques. Anticipatory approaches allow for detailed and comprehensive forward-looking ethical analyses. As they rely on information and descriptions about plausible or possible futures, they are to a degree uncertain and speculative. Even though foresight and forecasting methods often combine anticipatory with participatory approaches resulting in qualitative assumptions about certain aspects of possible futures, it is also possible – as in approaches like ethical risk analysis (defining, assessing, analysing and managing risks) and ethical risk-benefit analysis (based on the ratio of the risk of an action to its related potential benefits) – to use quantitative methods. Such methods can provide quantitative, ethically based assessments of risks and benefits of emerging technologies. At the same time, a quantitative determination of risks and potential benefits will often be contentious (Brey 2017).

The need for anticipation also puts forward problems for the most common method of policy advice, cost-benefit analysis (CBA). The reason for this is that CBA assumes that all possible significant consequences can be enumerated in advance and that probability, cost and benefit values can be attributed to them (Baer and Spash 2009). Economists have come up with some alternatives such as cost-effectiveness analysis (CEA), which sets a policy goal such as climate stabilisation that should then be achieved with minimal welfare loss; or cost-risk analysis (CRA), a hybrid approach between CBA and CEA (Neubersch et al. 2014). Especially forms of integrated assessment models (IAMs) that use CEA, explicitly renouncing problematic climate change damage functions, can be useful to gain a better insight of possible consequences of the deployment of certain CE options. IAMs represent the impacts of certain human activities on the climate system or terrestrial systems and on human activities and collect and summarise information from different fields of study. For example, they can provide us with information about the potential influences of different land-based CDR technologies on expected changes in food prices. IAMs are also able to evaluate portfolio approaches and can therefore give answers towards questions of the potential influence of one option in combination with other options. They can also show distributional effects on regions, social and economic sectors or groups in their data. The temporal and spatial distribution of benefits, burdens, and risks is of special interest for the normative assessment of CE options. Up to now, it is an open question whether all or some CE technologies would increase existing inequalities and historical injustices of climate change (Svoboda et al. 2011). This also holds true for long-term consequences. As it is widely discussed in the literature, some CE technologies could lead in their consequence to an increase of inequalities between generations as they might allow deferring risks and costs to future generations (Gardiner 2010; Ott 2012; Goes et al. 2011; Svoboda 2016).

This amounts not only to the need for ethically reflecting on the results put forward by IAMs, but also for a better understanding of the fact that they depend on assumptions about future developments of societies, economics and international relations. One should not believe that they are able to reveal the future and all potential effects and consequences of the use of a technology. They always need to rely on certain scenarios that are merely possible narratives about the future in general. In order to make the results of the economic analyses more comparable, various scenarios have been introduced in the scientific community, such as the Representative Concentration Pathways (RCPs) or the recently developed Shared Socio-economic Pathways (SSPs). Even though these standardised scenarios are used by economists, their influence on the results are often overlooked from the viewpoint of philosophy. For our normative assessment, we must take this into account and refer to the underlying assumptions of these scenarios when using information based on economic models. This would also require us to improve our understanding of the role and impact of these scenarios for the set-up and the results of the IAMs. Here one can refer back to the concept of explorative philosophy and the vision evaluation approach (see above).

Furthermore, like with any multi-criteria approach, we have to be aware of the intrinsically subjective elements in policy analysis. As described by Stirling (2006), 97) these ‘framing’ factors include “the nature and scope of the decision problem, the selection and definition of options, and the characterising and prioritising of evaluative criteria.” The development of such evaluative criteria is one of the main challenges for the evaluation of CE technologies. In order to evaluate different options, we need indicators that show us how well or poorly an option or set of options meets certain objectives, criteria or standards. This involves a translation process that starts with certain objectives, transfers them into specific criteria, which need then be translated into indicators showing the extent to which certain options meet or fail to meet different standards. The objectives we consider and the criteria or standards we develop cannot be set by science. This process is based on value judgements formulated by experts and/or the result of social or political processes. However, we can also use mixed approaches by using and adapting widely accepted goals and criteria.

The CE community – at least in Europe – has shown an increasing interest in the 17 Sustainable Development Goals (SDGs) which are discussed as possible starting points for the development of criteria and indicators for CE options. The use of the SDGs has a number of advantages. It can help to discard a purely individualist perspective on ethics as demanded in the paradigm shift by Timmermans and Blok (2018) for RRI by not only taking into account the interests but also the inputs from wider societal stakeholders also on a global level. The basic formulation of the SDGs rests itself on an inclusive process. As they have been adopted by almost 200 countries, they come with a high international legitimacy. Although the SDGs are not legally binding, governments are expected to assume responsibility based on the 2030 Agenda for Sustainable Development and to set national frameworks for achieving them. Over the next 15 years, the SDGs are expected to lead mobilisation efforts to end all forms of poverty, inequality and climate change. They are therefore expected to be known to policy-makers and to influence the debates on sustainability and climate change. In addition, the SDGs provide a list of 169 objectives that can serve as an approximation to some of the criteria and indicators needed for the evaluation of CE technologies.

Still, from a normative viewpoint we need to conduct in-depth philosophical research before using the SDGs and to look for possible conflicts between different objectives (Holden et al. 2017; Nilsson and Lucas 2013). For the development of criteria, one would also need to refer to indicators that are able to signal the (expected) transgression of certain thresholds as minimal standards that need to be fulfilled by any given option (Stelzer and Schuppert 2016). Such an approach enables the use of multiple thresholds functioning as guardrails that open up a certain range of options while also clearly indicating when an option is outside of this range. While guardrails allow the use of measurable quantities based on specified indicators, their multidimensionality enables us to take into account socio-ethical issues raised by the justice considerations mentioned above. They could also offer important answers towards the weighing of different goals and criteria which is a main challenge for the ethical evaluation of CE options.

## Conclusion

**Fig. 1:**
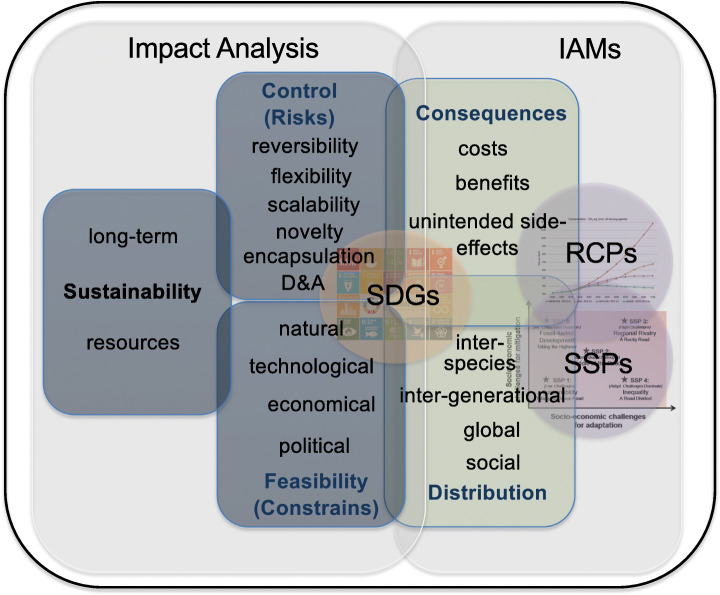
Evaluative-framework for CE options based on on socio-economic outcomes (including costs, benefits and unintended side-effects) and their distribution and their relation to issues of controllability, feasibility and sustainability

As argued in this paper, a TA based normative evaluative framework (see Fig. 1) could help to develop a more differentiated view on CE technologies that rests on interdisciplinary research and on more accurate criteria to inform our judgments on the normative permissibility of certain CE options. As pointed out, we are still in an early research stage, economy is not dominating considerations for societal or ethical values yet, stakeholder involvement up to now is limited and it may be too early to actively intervene into the research and development process to steer it in a certain direction, even if this is done in a democratic way by the wider public. While there is much room in the developed normative evaluative framework for the first two elements of the Stilgoe et al. (2013) RRI approach, anticipation and reflexivity, some aspects of the last two elements – inclusion and responsiveness – need not to be our primary concern, at least not for the moment. This, however, does not mean that it is not necessary to include societal and ethical concerns already at this early stage. The ethical issues are manifold, and early engagement of ethicists is of utmost importance. The approach offered can take up the manifold ethical issues, while at the same time transcending some limits of the dominant ethical discussion. To include the relevant interests of stakeholders as well as questions of global and intergenerational justice, one can link the normative evaluation of CE options to the SDGs. Also, epistemological challenges of uncertainty can be taken up by engaging in interdisciplinary research and with the results provided by impact analysis and IAMs. Even though we will never be able to foresee the future in detail, we can use standard scenarios to compare results from different analyses, models and studies. We therefore do not need to surrender due to an uncertainty that will always surround emerging technologies. What we need is a high-quality management of uncertainty (Funtowicz 1990) which should also be reflected in our understanding of research results and knowledge gaps. By considering the societal and ethical issues at stake, the envisioned evaluative framework can support us in enhancing our understanding and in guiding research and development processes of CE technologies. With this it gets closer to what we expect from output oriented RRI.

## Footnotes

1 More information on SPICE can be found under http://www.spice.ac.uk.

2 https://www.theguardian.com/environment/2012/may/16/geoengineering-experiment-cancelled.

3 Of course, there had been concerns about CE field tests also before SPICE, as expressed by United Nations Convention on Biodiversity in regard to their possible effects on biodiversity (http://www.cbd.int/climate/geoengineering/).

4 For more informaition see https://projects.iq.harvard.edu/keutschgroup/scopex.

5 For general arguments for and against public participation and dialogue see the section on inclusion in Stilgoe et al. (2013) as well as the overview of arguments in Noorman et al. (2017). For different justifications (normative, substantive, instrumental) for increased participation see Sterling (2006, 96). For a differentiated view on responsiveness on different levels see Sonck et al. (2017). As much of the discourse of RRI concerns publicly funded research it is not immediately obvious whether the arguments in favour of RRI are relevant to companies which shoulder a significant amount of research and innovation activities (Blok and Lemmens 2015). Even though privately funded organizations have a different incentive structures RRI seems relevant to companies as it could help to by strengthening ties with stakeholders including customers, to improve corporate image, to facilitate alignment with societal and regulatory expectations and requirements, and even to enhance employee satisfaction. For the constrains and opportunities of RRI in companies see Blok et al. (2015), the maturity model by Stahl et al. (2017) and the Datashare case-study by Noorman et al. (2017).

6 Wicked problems stand opposite to ‘tamed problems’ because they are highly complex and not amenable for definitive solutions. For such problems, solutions are often not true-or-false but better or worse and – one has to add for challenges to the environment: more or less sustainable (Rittel and Webber 1973).

7 I thank the anonymous reviewer for raising this question.

8 EIA has been developed especially with regard to information technology (with a focus also on stakeholder involvement). A prominent example is Wright (2010) EIA framework.
